# LncRNA affects epigenetic reprogramming of porcine embryo development by regulating global epigenetic modification and the downstream gene *SIN3A*

**DOI:** 10.3389/fphys.2022.971965

**Published:** 2022-09-16

**Authors:** Daoyu Zhang, Yongfeng Zhou, Rong Huang, Yanhui Zhai, Di Wu, Xinglan An, Sheng Zhang, Lijing Shi, Qi Li, Xiangjie Kong, Hao Yu, Ziyi Li

**Affiliations:** ^1^ Key Laboratory of Organ Regeneration and Transplantation of Ministry of Education, First Hospital, Jilin University, Changchun, China; ^2^ Department of Emergency Medicine, First Hospital, Jilin University, Changchun, China; ^3^ College of Animal Science, Jilin University, Changchun, China

**Keywords:** lncRNAs, histone modification, DNA methylation, *SIN3A*, porcine embryonic development

## Abstract

The study of preimplantation development is of great significance to reproductive biology and regenerative medicine. With the development of high-throughput deep sequencing technology, it has been found that lncRNAs play a very important role in the regulation of embryonic development. In this study, key lncRNAs that regulate embryonic development were screened by analyzing the expression pattern of lncRNAs in porcine *in vivo* fertilization (IVV) embryos. By knocking down lncRNA expression in *in vitro* fertilization (IVF) embryos, we investigated its function and mechanism of regulating embryonic development. The results showed that the expression pattern of lncRNA was consistent with the time of gene activation. The lncRNAs were highly expressed in the 4-cell to blastocyst stage but barely expressed in the oocytes and 2-cell stage. So we speculated this part of lncRNAs may regulate gene expression. The lncRNA LOC102165808 (named *lncT* because the gene near this lncRNA is *TFAP2C*) was one of them. The knockdown (KD) of *lncT* inhibited embryonic development, resulting in decreased H3K4me3, H3K4me2, and H3K9me3, and increased DNA methylation. Meanwhile, RNAseq showed *SIN3A* was the top decreased gene in *lncT-*KD embryos. There was a severe blastocyst formation defect in *SIN3A-*KD embryos. Both *lncT* and *SIN3A* could affect *NANOG* and induce more cell apoptosis. In conclusion, the knockdown of *lncT* inhibits embryonic development by regulating H3K4me3, H3K4me2, DNA methylation, pluripotency gene, and apoptosis, and *SIN3A* is one of the downstream genes of *lncT* in regulating embryonic development.

## Introduction

With the development of high-throughput deep sequencing technology, it has been found that lncRNAs play a very important role in the regulation of animal embryonic development. Wu et al. (2019) reported that *MALAT-1* plays a key role in the embryonic nervous system development of zebrafish. Wang et al. (2016) indicated that linc*GET* is essential for correct ZGA processes and further promotes the cleavage of two-cell embryos *via* regulating transcription and RNA alternative splicing in mice. In the goat embryos, the knockdown of the lncRNA *TCONS_00460156* significantly decreases the developmental rate (Deng et al., 2019). Deng et al. (2021) showed that lnc*_3712* impedes nuclear reprogramming *via* repressing *KDM5B*, and the microinjection of siRNA against lnc_*137* causes development arrests (Deng et al., 2018). However, different from coding RNAs, lncRNAs are less conserved among species (Kern et al., 2018), and studies on lncRNA in pig embryo development are scarcer. It is reported that lncRNA_*2193* regulates meiosis through global epigenetic modification and cytoskeleton organization in pig oocytes but does not affect the cleavage and blastocyst rates of parthenotes (Yang et al., 2020). Thus, more research is needed to explore the mechanisms of lncRNAs in embryonic development.

Epigenetic modification, such as histone modification and DNA methylation, plays an important role during early embryonic development. In previous studies, H3K4me3 (Dahl et al., 2016) (Liu et al., 2016), H3K9me3 (Wang et al., 2018), and H3K27me3 (Liu et al., 2016) have been identified as epigenetic barriers during nuclear reprogramming in mice. In multicellular eukaryotes, H3K4me3 is predominantly localized at gene promoter regions, centered on the transcriptional start sites (Zardo, 2021). H3K4me3 deposited at the promoter-proximal and gene body regions facilitates transcription elongation and mRNA maturation (Foroozani et al., 2021). H3K4me3 might function as a transcription “booster” by promoting RNAP II loading onto promoters or its release into elongation (Muramoto et al., 2010). The loss of H3K4me3 causes the retention of RNAP II at promoters of developmental and stress genes and stalls their upregulation (Ardehali et al., 2011). In embryo development, H3K4me3 leads to gene transcription from developing gametes to post-implantation embryos (Zhang et al., 2016). A previous study reported that the downregulation of H3K4me3 in full-grown oocytes by overexpression of the H3K4me3 demethylase KDM5B was associated with defects in genome silencing (Zhang et al., 2016).

During the regulation of epigenetic modification processes, DNA methylation and histone modification interact with each other. DNA methylation is the earliest and most extensively studied epigenetic modification, which takes the CpG island as the central link (Zhou et al., 2021). Recent studies have indicated that methylation of CpG islands is related to the methylation status of H3K4 (Zardo, 2021), and the levels of methylated H3K4 (H3K4me3) tend to be inversely correlated with DNA methylation (Okitsu Cindy and Hsieh, 2007). DNA methylation is mainly established and maintained by DNA methyltransferases (DNMTs) that transfer a methyl group from S-adenyl methionine (SAM) to the fifth carbon of a cytosine residue to form 5mC (Zhou et al., 2021) (Chen and Zhang, 2020), while the active removal of methylation marks relies on the activity of ten-eleven translocation (TET) enzymes and thymine DNA glycosylase (TDG) (Parry et al., 2021). After fertilization, including the deletion of most methylation marks inherited from the gametes and the subsequent establishment of the embryonic methylation pattern, they show dynamic changes during development (Zeng and Chen, 2019). However, the epigenetic modification of lncRNA needs to be further studied.

Pigs are important livestock in agriculture and valuable animal models for xenotransplantation, somatic cell nuclear transplantation, stem cell therapy, and basic biology research (Zhang et al., 2018). Fertilization is followed by complex changes in cytoplasmic composition and extensive chromatin reprogramming, which results in the abundant activation of the totipotent embryonic genome at embryonic genome activation. It is commonly believed that embryonic genome activation occurs during the 4-cell stage in the porcine embryo (Østrup et al., 2013; Zhang et al., 2022). In this study, key lncRNAs that regulate embryo development were screened by analyzing the expression pattern of lncRNA in porcine IVV embryos. By knocking down lncRNA expression in IVF embryos, we investigated its function and mechanism for regulating embryonic development. This study fills in the deficiency of lncRNA research in porcine embryo development and provides theoretical support for biomedical and animal husbandry production.

## Materials and methods

### LncRNA isolation and analysis

The RNAseq data on IVV embryos we used are the data we have uploaded to NCBI previously (https://submit.ncbi.nlm.nih.gov/subs/sra/SUB10711406/PRJNA 783716), under accession IDs: MII_1 (SRR17041081), MII_2 (SRR17041080), MII_3 (SRR17041074), IVV_2C_1 (SRR17041073), IVV_2C_2 (SRR17041072), IVV_2C_3 (SRR17041071), IVV_4C_1 (SRR17041070), IVV_4C _2 (SRR17041069), IVV_4C_3 (SRR17041068), IVV_8C_1 (SRR17041067), IVV _8C_2 (SRR17041079), IVV_8C_3 (SRR17041078), IVV_Bla_1 (SRR17041077), IVV_Bla_2 (SRR17041076), and IVV_Bla_3 (SRR17041075). LncRNAs were isolated according to non-coding annotations of pigs in the ALDB database (http://202.200.112.245/aldb/); the specific data are shown in Supplementary Table S1. Differentially expressed genes (DEGs) were analyzed by IDEP, and heat maps were constructed. The pathway enrichment of DEGs was obtained through WebGestalt (http://www.webgestalt.org/option.php). The bubble plot of pathway enrichment was constructed by BioLadder (https://www.bioladder.cn/web/#/pro/cloud); the specific data are shown in Supplementary Table S2. The target genes were predicted by the ALDB database, the information on lncRNAs and coexpressed genes is shown in Supplementary Table S3, and the Venn diagram was constructed by FunRich version 3.1.3.

### Antibodies and chemicals

Antibodies used are as follows: H3K4me2 (Abcam; ab7766, diluted 1: 200), H3K4me3 (Abcam; ab8580, diluted 1: 200), H3K9me3 (Abcam; ab8898, diluted 1: 200), 5mC (Eurogentec; BI-MECY-0100, diluted 1:100), 5hmC (Active Motif; 39,769, diluted 1:100), DNMT1 (Invitrogen, MA5-16169, diluted 1:10), Alexa Fluor 488 goat anti-rabbit (Invitrogen, A-11008, diluted 1: 200), and/or Alexa Fluor 488/594 goat anti-mouse (Invitrogen, A32723/A-11020, diluted 1: 200) antibodies. All the chemicals used to culture were purchased from Sigma-Aldrich (St. Louis, MO, United States), unless otherwise stated.

### The collection and *in vitro* maturation (IVM) of porcine oocytes

The pig ovaries we used were taken from the same local slaughterhouse (Changchun Huazheng, Jilin, China); the pigs are the cross of Landrace boar and Large White sow. The ovaries were transported to the laboratory within 2 h. They were kept in 0.9% NaCl supplemented with 200 IU/ml penicillin and streptomycin at 35–36.5°C.

The follicular fluid containing cumulus–oocyte complexes (COCs) from 3–6 mm ovarian follicles was aspirated using an 18-gauge needle. COCs with at least three layers of cumulus cells were selected, washed three times in a manipulation fluid (TCM-199 supplemented with 0.1% polyvinyl alcohol), and then cultured in the media of IVM. Approximately, every 200 COCs were cultured in a 1 ml drop of maturation medium (TCM-199 supplemented with 10 μg/ml epidermal growth factor, 0.5 μg/ml porcine luteinizing hormone, 0.5 μg/ml porcine follicle-stimulating hormone, 26 mM sodium bicarbonate, 3.05 mM glucose, 0.91 mM sodium pyruvate, 0.57 mM cysteine, 0.1% PVA, 10% fetal calf serum, 75 mg/ml penicillin G, and 50 mg/ml streptomycin) for 22–24 h at 38.5°C, 5% CO_2_, and 95% air. Then, they were transferred to a hormone-free maturation medium (the formula is consistent with the previous maturation medium without 10 μg/ml epidermal growth factor, 0.5 μg/ml porcine luteinizing hormone, and 0.5 μg/ml porcine follicle-stimulating hormone) for 20 h at 38.5°C, 5% CO_2_, and 95% air. Then, cumulus cells were removed from oocytes with a manipulation fluid supplemented with 0.2% hyaluronidase. The oocytes with polar body 1 (PB1) were considered matured and used for the following experiments.

### Microinjection of siRNAs

siRNA was injected using the microinjection meter (Eppendorf, FemtoJet 4i, United States). A measure of 5–10 pL of 25 nM *lncT* siRNA, *SIN3A* siRNA, or NC siRNA was injected into MⅡ oocytes, and then, the oocytes were incubated with siemens in PGM (porcine gamete medium: 100 ml water with 0.6313 g NaCl, 0.07456 g KCl, 0.00477 g KH_2_PO_4_, 0.00987 g MgSO_4_·7H_2_O, 0.2106 g NaHCO_3_, 0.07707 g CaC_6_H_10_O_6_·5H_2_O, 0.0187 g D-Glucose, 0.3 g PVA, 0.00242 g cysteine, 0.04504 g C_7_H_8_N_4_O_2_, 0.0022 g C_3_H_3_NaO_3_, and 100 μl/ml penicillin/streptomycin). The *lncT* siRNA, *SIN3A* siRNA, and NC siRNA were designed and synthesized by Sangon Biotech (Shanghai, China). The sequences were listed as follows:

*lncT*: sense: CCA​GAU​GAG​AUG​GUG​AUA​ATT,
anti-sense: UUA​UCA​CCA​UCU​CAU​CUG​GTT;
*SIN3A*: sense: CCA​AGU​GAA​GCU​ACA​GUU​UTT,
anti-sense: AAA​CUG​UAG​CUU​CAC​UUG​GTT;
NC: sense: UUC​UCC​GAA​CGU​GUC​ACG​UTT,
anti-sense: ACG​UGA​CAC​GUU​CGG​AGA​ATT.

### *In vitro* fertilization (IVF) of oocytes

Fresh semen was collected from the Jilin University pig farm. Density gradient centrifugation was used to wash them. In brief, percoll was configured in the concentration of 90% and 45%, 2 ml of semen was added and centrifuged at 300 g for 20 min, and the supernatant was removed after centrifuge. Then, 4 ml of DPBS was added and centrifuged at 300 g for 10 min. The sperm were resuspended with PGM. Sixty denuded matured oocytes were each cultured in 400 μl PGM with a final sperm concentration of 1.6 × 10^5^—5.0×10^5^ sperm/ml, at 38.5°C and 5% CO_2_ for 5–6 h. After washing off the adherent sperm, the fertilized oocytes were transferred to PZM-3 (Yoshioka et al., 2002).

### RNA-seq

The smart-seq2 method was used to amplify each sample (6–8 embryos for each group), according to the manufacturer’s instructions. The RNA concentration of library was measured by using a Qubit 2.0 Fluorometer (Life Technologies, CA, United States). The Agilent Bioanalyzer 2100 system was used to assess the insert size, and the quality of the amplified products was evaluated according to the detection results. The amplified product cDNA was used as the input for the library construction of transcriptomes. After the library construction, the Agilent Bioanalyzer 2100 system was used to assess the insertion size, and the TaqMan fluorescence probe of an AB Step One Plus Real-Time PCR system (library valid concentration >10 nM) was used to quantify the accurate insertion size. Clustering of the index-coded samples was performed using a cBot cluster generation system and the HiSeq PE Cluster Kit v4-cBot-HS (Illumina). Then, the libraries were sequenced by Zhejiang Annoroad Biotechnology (Beijing, China) on an Illumina platform, and 150-bp paired-end reads were generated. STAR was used to compare transcriptome data and Cufflinks for quantitative splicing. DEmRNAs were identified by the IDEP website (http://bioinformatics.sdstate.edu/idep/). Genes with false discovery rates (FDRs) ≤ 0.05, |log2FC| ≥ 1.5, and *p*-value ≤ 0.01 were selected as candidate genes, and the specific data are shown in Supplementary Table S4.

### RNA isolation and qPCR

Total RNA was extracted, and complementary DNA (cDNA) was synthesized with the SuperScript™ IV CellsDirect™ cDNA Synthesis kit (11750350, Invitrogen, United States), following the manufacturer’s instructions. The qPCR was performed with FastStart Essential DNA Green Master (06924204001, Roche, United States) *via* a StepOnePlus Real-Time PCR system. Primers sequences were listed as follows:

*lncT*: forward-5′-GGTCACTTGGCAGAGATGCT-3′
reverse-5′-GTAGAGAGCGGAGAACGTCG-3’;
*SIN3A*: forward-5′-CTCTCCCACCATACGCATCC-3′,
reverse-5′-CTCAGTCAACGCTGGAGTGT-3’;
*NANOG*: forward-5′- CAG​GGT​GGT​GAA​GTG​AGG​G-3′,
reverse-5′-CCCCGAAGCATCCATTTCC-3’;
*GAPDH*: forward-5′-AGGTCGGAGTGAACGGATTTG-3′,
reverse-5′- CCA​TGT​AGT​GGA​GGT​CAA​TGA​AG -3’.

The results were analyzed by the 2^−ΔΔ*C*T^ method. The qPCRs were all repeated three times.

### Immunofluorescence (IF) staining

A total of 5–8 embryos of IVF were washed with PBS containing 0.1% polyvinylpyrrolidone (PVP). The zona pellucida of embryos was dissolved in an acidic Tyrode solution (pH 2.5). After washing in PBS-PVP, embryos were fixed with 4% paraformaldehyde for 30 min in the dark. After being washed in PBS-PVP, embryos were permeabilized with 0.2% Triton X-100/PBS (v/v) for 20 min and then blocked with 2% BSA/PBS for 1 h. For 5mC/5hmC staining, the embryos were treated with 4N-HCl and Tris-HCl for 30 min each before BSA blocking. Embryos were incubated with primary antibodies at 4°C overnight. After being washed in PBS-PVP, embryos were stained with a secondary antibody at 37°C for 2 h in dark. Then, DNA was stained with 10 μg/ml DAPI for 15 min. All samples (DNMT1 excepted) were observed under a Nikon Eclipse Ti-U microscope equipped with appropriate filters (Nikon, Tokyo, Japan) after mounting. Color images were captured using a DS-Ri2 CCD camera (Nikon, Tokyo, Japan) and an analysis software application (NIS-Elements BR; Nikon, Tokyo, Japan). The same exposure times and microscope settings were used for all captured images. Evaluation of the fluorescence intensity of individual images was performed by ImageJ software (National Institutes of Health, Bethesda, MD). The cytoplasmic background fluorescence intensity was measured as an average intensity level within the cytoplasmic area. Thereafter, the correction for the cytoplasmic background was carried out, and the background-subtracted images were used for further analysis (Zaitseva et al., 2007). The DNMT1 immunofluorescence staining images were obtained by confocal microscopy (ZEISS, Examiner, Z1/LSM880). At least five embryos at each development stage were analyzed.

### Detection of apoptosis of the blastocysts

After the removal of the zona pellucida, fixation by 4% paraformaldehyde, permeabilization by 1% Triton-X 100, and blocked by 1% BSA, the blastocysts were incubated with a term deoxynucleotidyl transferase dUTP nick-end labeling (TUNEL) solution from the *In Situ* Cell Death Detection Kit (Roche, Mannheim, Germany) at 37°C for 1 h in darkness. The blastocysts were washed with PBS-PVP three times and stained with DAPI (10 μg/ml) for 15 min. The stained blastocysts were mounted between a cover slip and a glass slide and observed under a fluorescence microscope (Nikon, Tokyo, Japan), with 5–8 blastocysts per group.

### Statistical analysis

Data are presented as the means ± SEMs. The experiments were repeated at least two times in triplicate. Statistical analysis was performed by GraphPad Prism 6.01 (GraphPad Software, United States). A chi-squared test was performed to analyze the ratio of the developmental capacity and efficiency of the control and *SIN3A* KD embryo data between the two groups. *p < 0.05, **p < 0.01, and ***p < 0.001 were considered statistically significant.

## Results

### Differentially expressed lncRNAs in IVV embryos

**FIGURE 1:**
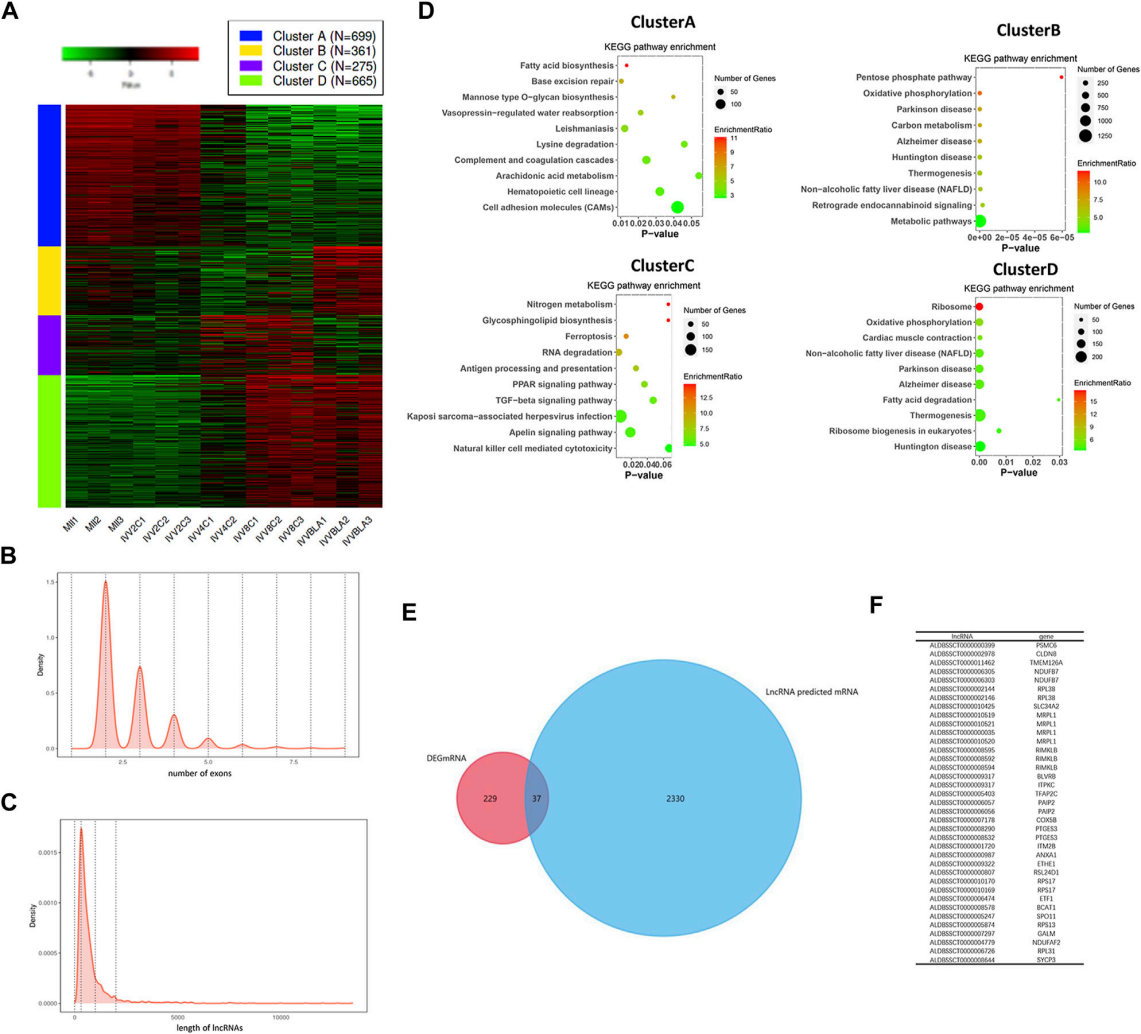
LncRNA expression pattern in embryos of IVV. **(A)** Heatmap of lncRNAs in embryos; the colors in the map displayed the relative normalized values; blue indicated the lowest expression, and red indicated the highest expression. **(B)** Average number of exons of the lncRNAs. **(C)** Average length of the lncRNAs. **(D)** KEGG pathway enrichment of the DElncRNAs. The size of the dot in the bubble plot indicated the number of genes, and the color indicated the enrichment ratio. **(E)** Venn diagram of the target genes lncRNA predicted and coexpression genes. **(F)** Thirty-seven main lncRNAs and their predicted genes.

To explore the differentially expressed lncRNAs in IVV embryos, a heatmap was structured. As shown in Figure 1A, there were 2,000 different expressed lncRNAs, and 699 lncRNAs presented decreased expression when embryos developed to the 4-cell stage, 361 lncRNAs were upregulated in the blastocyst stage, 275 lncRNAs were upregulated in 4-cell and 8-cell stages, and 665 lncRNAs were increased in 8-cell and blastocyst stages (FDR<0.05 fold change>2). The expression pattern of lncRNA was consistent with the time of gene activation, and we speculated that this part of lncRNA played a role in regulating gene expression. The average number of exons of the lncRNAs was 2.5 (Figure 1B), and the average length of the lncRNAs was 310bp (Figure 1C), which was consistent with the characteristics of lncRNAs. To study the functions of the DElncRNAs, Kyoto Encyclopedia of Genes and Genomes (KEGG) enrichment analysis was carried out. As shown in Figure 1D, genes in cluster A were mainly enriched in fatty acid biosynthesis, base excision repair, and cell adhesion molecule pathways. Cluster B was enriched in the pentose phosphate pathway and oxidative phosphorylation carbon metabolism. Genes in cluster C were enriched in nitrogen metabolism, glycosphingolipid biosynthesis, and RNA degradation pathways. Also, cluster D was enriched in ribosome, oxidative phosphorylation, fatty acid biosynthesis, etc. To select the candidate lncRNA for regulating embryonic development, we predicted the targeting genes of these lncRNAs, which are upregulated from the 4-cell stage, and coexpression was analyzed with the mRNAs with the same characteristics. As shown in Figures 1E,F, a total of 37 lncRNAs were selected. These results suggested that the highly expressed lncRNAs have the potential role in embryonic development, and the 37 lncRNAs were the candidate genes.

### The knockdown (KD) of *lncT* inhibited embryonic development

**FIGURE 2:**
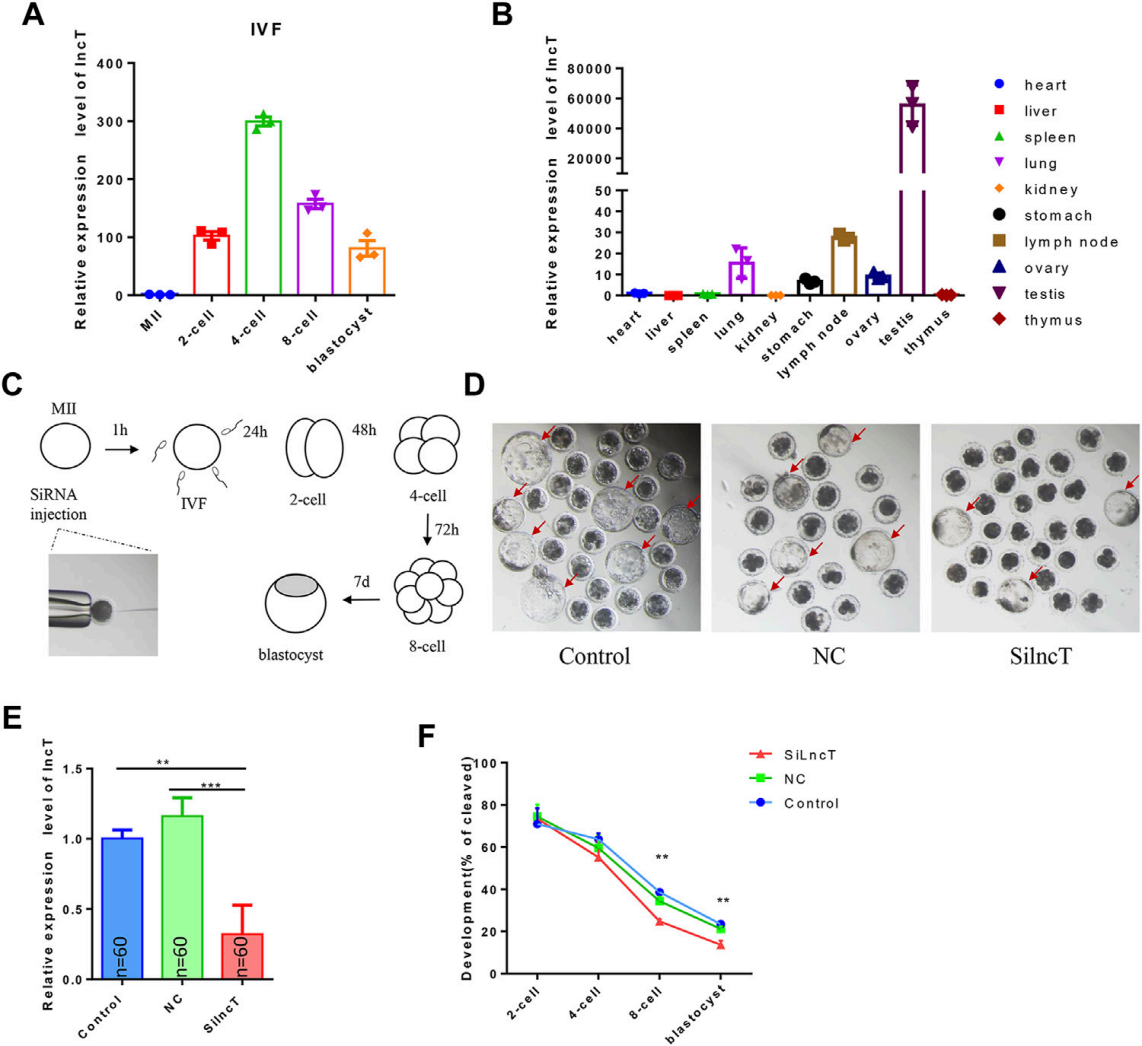
Knockdown of *lncT* inhibited embryonic development. **(A)** Expression of *lncT* in IVF embryos. The experiment was independently repeated three times. **(B)** Expression of *lncT* in porcine different organs. **(C)** Flow chart of interfering RNA injection and observation of embryonic development of IVF. **(D)** Three images were the blastocysts of control, NC, and *lncT*-KD groups, respectively. **(E)** qPCR analysis of *lncT* expression of control, NC, and *lncT*-KD groups after siRNA injection 48 h (2-cell stage, *n* = 60 per group). The experiment was independently repeated three times. **(F)** Embryonic development rate in 2-cell, 4-cell, 8-cell, and blastocyst stages; **p < 0.01.

Since lncT (ALDBSSCT0000005403 in Figure 1F) has a single product when we use PCR to verify whether lncT exists in the pig genome, we chose lncT to conduct experiments to verify its role in embryos (Supplementary Figure S1). The total length of *lncT* is 2038bp, and the whole sequence is shown in Supplementary Table S5. It contains a polyA tail (Supplementary Figure S1). We detected its expression in IVF embryos, as shown in Figure 2A, and *lncT* was upregulated in 4-cell and 8-cell stages. Meanwhile, to explore the expression characteristics of *lncT*, we detected its expression in other organs, as shown in Figure 2B, and it was highly expressed in two germ organs, the testis and ovary. To investigate the functions of *lncT*, we knocked down the expression of *lncT* in embryos of IVF *via* the injection of small interfering RNAs (siRNAs) against it. As shown in Figure 2C, we injected siRNA or NC into MⅡ oocytes, and then investigated its effect on embryonic development. As shown in Figure 2D and Table 1, no difference was observed in the percentage of the blastocyst in the injected NC group (20.4 ± 0.95%) compared to the control group (22.92 ± 1.97%), suggesting that there was no significant effect on the NC group; thus, we will not inject NC in future experiments. However, the percentage of blastocysts in the *lncT*-KD group (13.65 ± 0.82%) was significantly decreased when compared to the control group (**p < 0.01). As expected, *lncT* was decreased in *lncT*-KD embryos compared to NC or the control group (***p < 0.001) (Figure 2E). Meanwhile, we also observed that the embryonic development difference was significant from the 8-cell stage (**p < 0.01) (Figure 2F). These data indicated that the knockdown of *lncT* inhibited porcine embryonic development.

**TABLE 1:** Development of porcine IVF embryos in control, NC, and Si-lncT groups. a, b Values with different superscript letters within a column differ significantly (*p* < 0.05). The experiment was replicated at least three times.

	Experiment (n)	Embryo (n)	2-cell stage (Mean ± SEM%)	4-cell stage (Mean ± SEM%)	8-cell stage (Mean ± SEM%)	Blastocyst (Mean ± SEM%)
Control	4	445	70.95 ± 3.25	63.7 ± 1.24	35.59 ± 2.00^a^	22.92 ± 1.97^a^
NC	3	283	74.42 ± 2.50	59.5 ± 2.71	34.35 ± 0.96^a^	20.4 ± 0.95^a^
Si-lncT	4	373	73.46 ± 2.20	55.1 ± 3.58	24.77 ± 0.52^b^	13.65 ± 0.82^b^

### Abnormal transcriptional reprogramming in *lncT*-KD embryos

**FIGURE 3:**
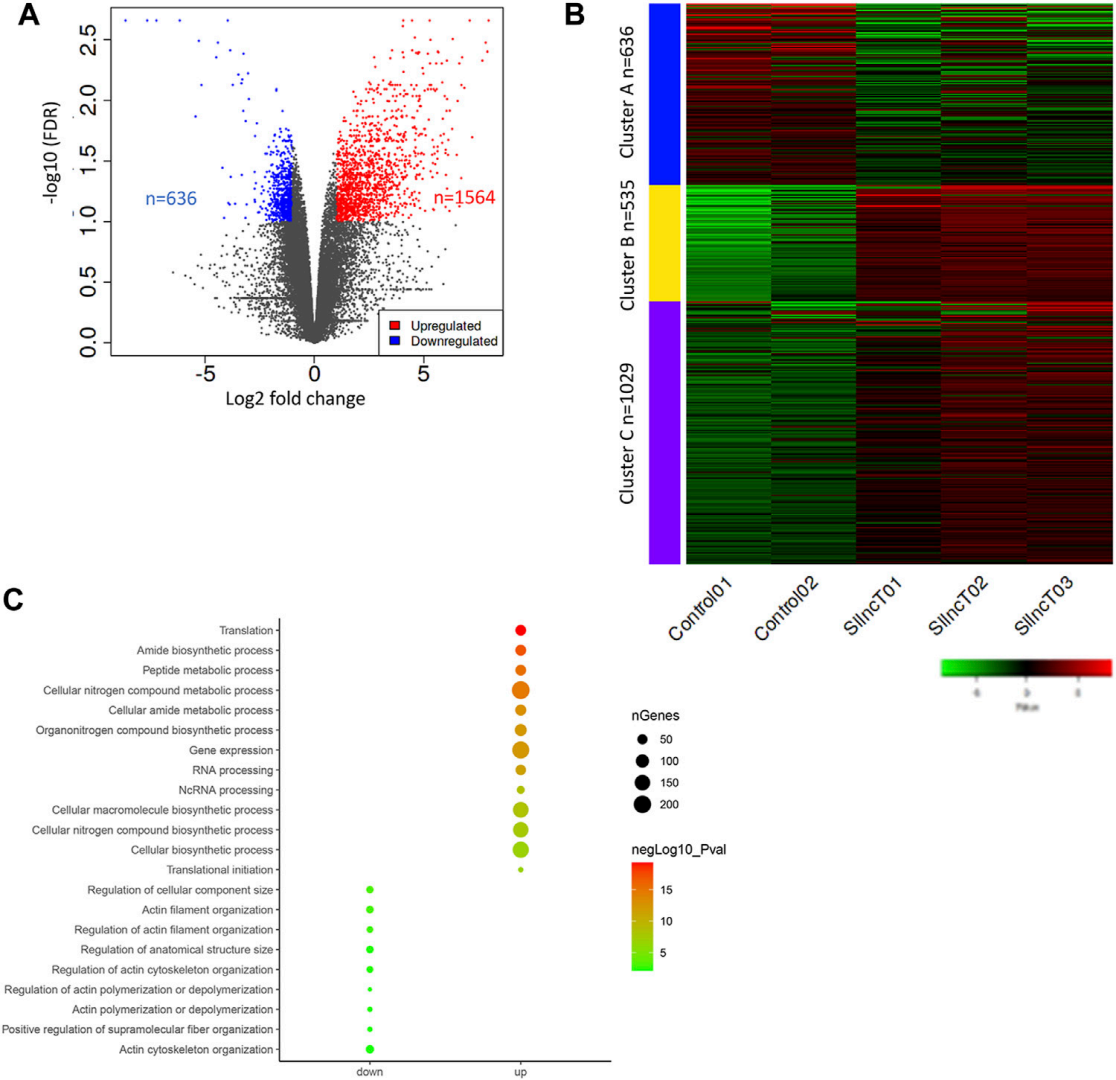
Abnormal transcriptional reprogramming in *lncT*-KD embryos. **(A)** Volcano plot of differentially expressed genes between the control group and *lncT*-KD group. Each point represented one gene; the red points indicated the upregulated genes, and blue points indicated the downregulated genes, FDR<0.1, fold change>2. **(B)** Heatmap of gene expression differences in the control group and *lncT*-KD group. **(C)** Pathway enrichment of upregulated genes and downregulated genes.

In order to study the mechanism of *lncT* in embryonic development, we collected embryos at the 4-8-cell stage, according to the arrest period of cleavage for transcriptome sequencing. As shown in Figure 3A, there were 636 downregulated genes and 1,564 upregulated genes after siRNA injection (FDR<0.05, logFC>2). The heatmap showed the transcriptome profile of the control group and *lncT*-KD group embryos (Figure 3B). Cluster A was the downregulated gene corresponding with the functions of regulation of cellular components, actin filament organization, and anatomical structure size (Figure 3C), while cluster B and -C were the upregulated genes corresponding with the functions of translation, the amide biosynthetic process, cellular nitrogen compound metabolic process, gene expression, and cellular macromolecule biosynthetic process (Figure 3C). These data revealed that the adverse effects in embryo development after siRNA injection may be due to abnormal gene expression.

### Changes in epigenetic modification levels were induced in *lncT*-KD embryos

**FIGURE 4:**
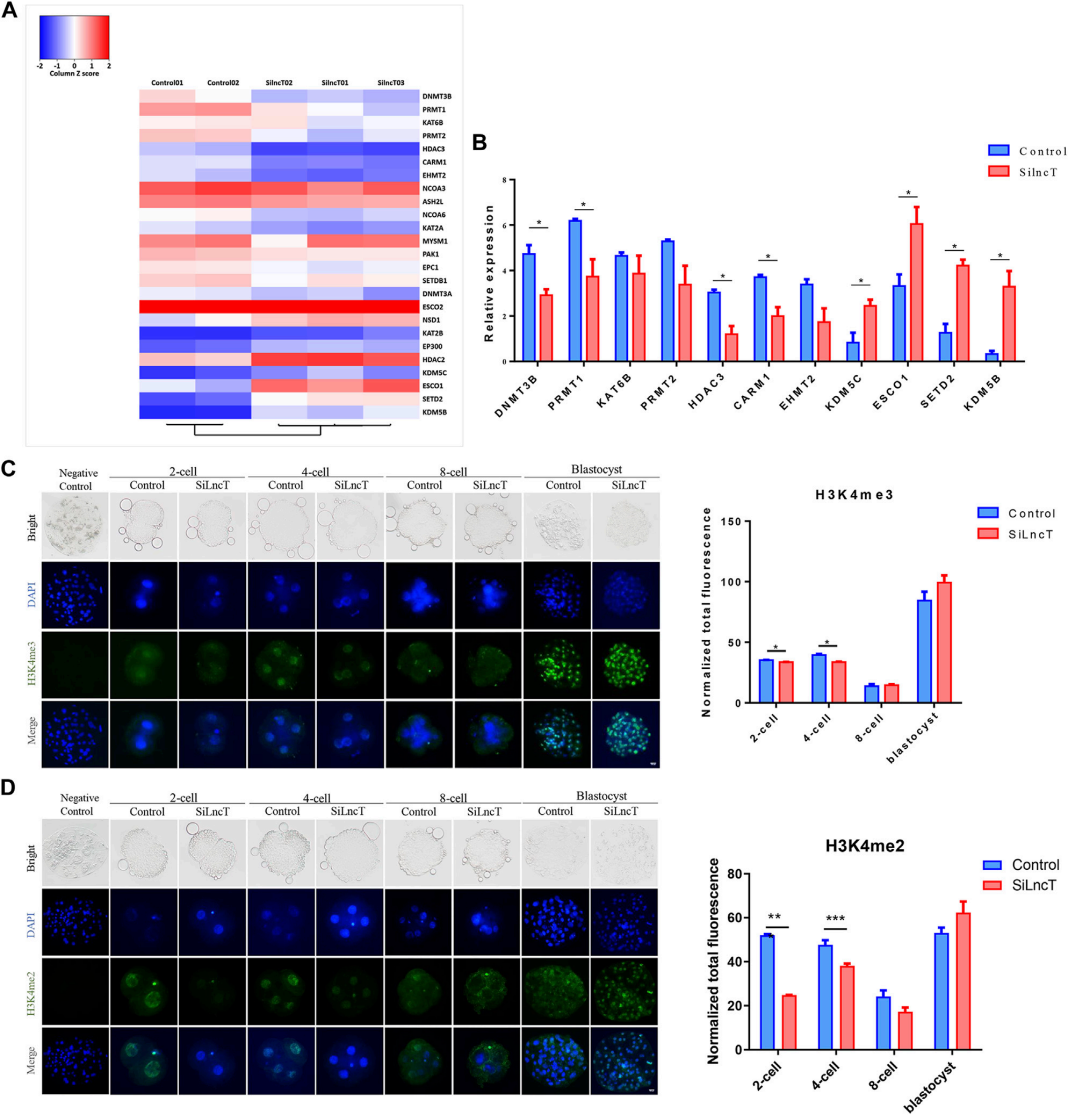
Changes in epigenetic modification levels were induced in *lncT*-KD embryos. **(A)** Heatmap of main epigenetic genes in the control group and *lncT*-KD group. The color scale bar was shown at the top of the heatmap. Blue indicated the lowest expression, and red indicated the highest expression. **(B)** Significantly changed epigenetic genes, *p < 0.05. The experiment was independently repeated three times. **(C)** Immunofluorescence of H3K4me3 in 2-cell, 4-cell, 8-cell, and blastocyst stages. The nuclei (blue) were stained with DAPI. Fluorescence intensity analysis was in the right of the image, *p < 0.05. The experiment was repeated three times independently with 5–8 embryos/stage/group/replicate. Negative control embryos were stained only with the secondary antibody. **(D)** Immunofluorescence of H3K4me2 in 2-cell, 4-cell, 8-cell, and blastocyst stages. The nuclei (blue) were stained with DAPI. Fluorescence intensity analysis was in the right of the image, ***p < 0.001. The experiment was repeated three times independently with 5–8 embryos/stage/group/replicate. Negative control embryos were stained only with the secondary antibody.

**FIGURE 5:**
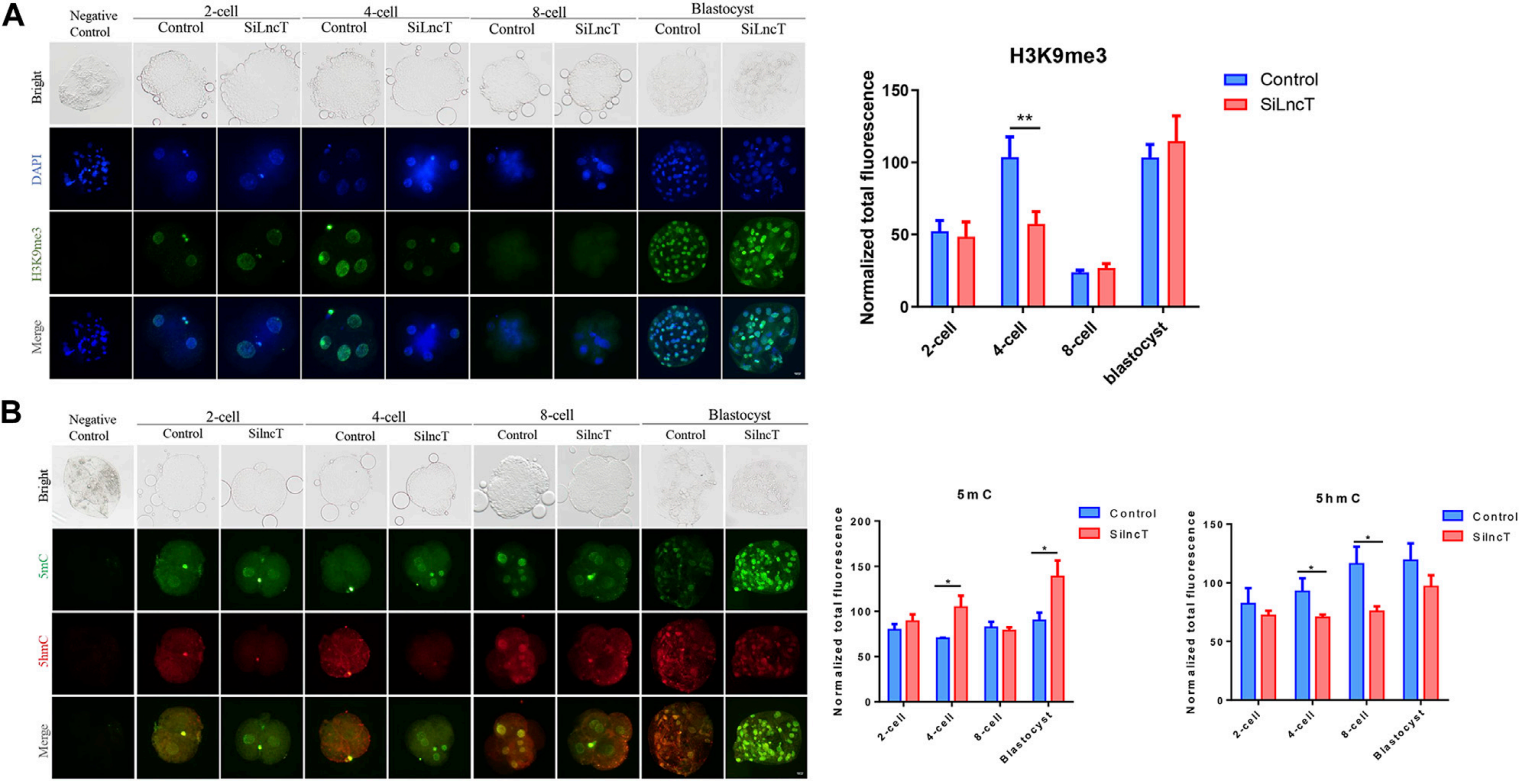
Changes in H3K9me3 and 5mC/5hmC. **(A)** Immunofluorescence of H3K9me3 in 2-cell, 4-cell, 8-cell, and blastocyst stages. The nuclei (blue) were stained with DAPI. Fluorescence intensity analysis was in the right of the image, **p < 0.01. The experiment was repeated three times independently with 5–8 embryos/stage/group/replicate. Negative control embryos were stained only with the secondary antibody. **(B)** Immunofluorescence of 5mC/5hmC in 2-cell, 4-cell, 8-cell, and blastocyst. The green indicated 5mC, and the red indicated 5hmC. Fluorescence intensity analysis was in the right of the image, *p < 0.05. The experiment was repeated three times independently with 5–8 embryos/stage/group/replicate. Negative control embryos were stained without treatment with 4N-HCI and Tris-HCI.

Next, we focused on the epigenetic modification genes in the transcriptome, as shown in Figure 4A, and the heatmap showed the differentially expressed genes between the two groups. As shown in Figure 4B, *DNMT3B*, *PRMT1*, *HDAC3*, and *CRAM1* were significantly downregulated (*p < 0.05), while *KDM5C*, *ESC O 1*, *SETD2*, and *KDM5B* were significantly upregulated (*p < 0.05) in *lncT*-KD embryos. Thus, we detected the expression of H3K4me3, H3K4me2, H3K9me3, and 5mC/5hmC during embryonic development. As shown in Figure 4C, the expression level of H3K4me3 was downregulated when *lncT* siRNA injection was injected in 2-cell and 4-cell stages (*p < 0.05). Also, H3K4me2 had a similar expression trend after *lncT* siRNA injection (***p < 0.001) (Figure 4D). The expression of H3K9me3 had no significant difference between the control and *lncT*-KD group in 2-cell, 8-cell, and blastocyst stages, but it was downregulated in the 4-cell stage (**p < 0.01) (Figure 5A). When we detected the expression of 5mC/5hmC, we observed that the 5mC modification was slowly lost during embryonic development, but it was always present in the *lncT*-KD embryos (*p < 0.05) (Figure 5B). Taken together, these results revealed that the knockdown of *lncT* affects epigenetic modification, and these effects were at the 4-cell stage.

### *SIN3A* was one of the downstream genes of *lncT* in regulating embryonic development

**FIGURE 6:**
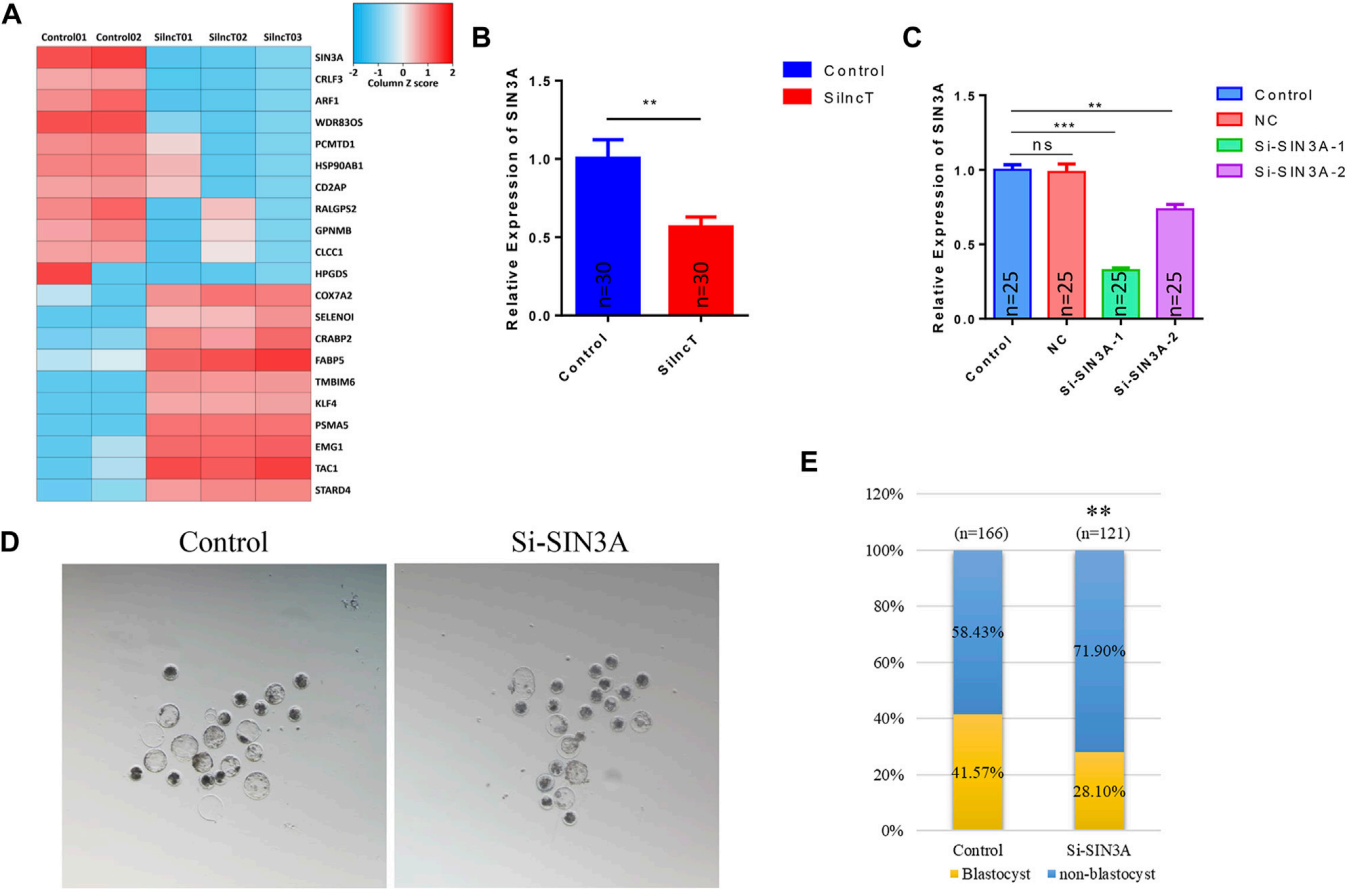
*SIN3A* was one of the downstream genes of *lncT*. **(A)** Heatmap of the top 10 upregulated genes and downregulated genes. The color scale bar is shown at the top of the heatmap. Blue indicated the lowest expression, and red indicated the highest expression. **(B)** qPCR analysis of *SIN3A* expression in the *lncT*-KD group (4-cell stage, *n* = 30 each group), **p < 0.01. The experiment was independently repeated three times. **(C)** Interference rate of siRNAs in embryos (4-cell stage, *n* = 25 in each group). The experiment was independently repeated three times. **(D)** Images were the blastocysts of control and *SIN3A*-KD groups, respectively. **(E)** Blastocyst rate in the control group and *SIN3A*-KD group, **p < 0.01.

**FIGURE 7:**
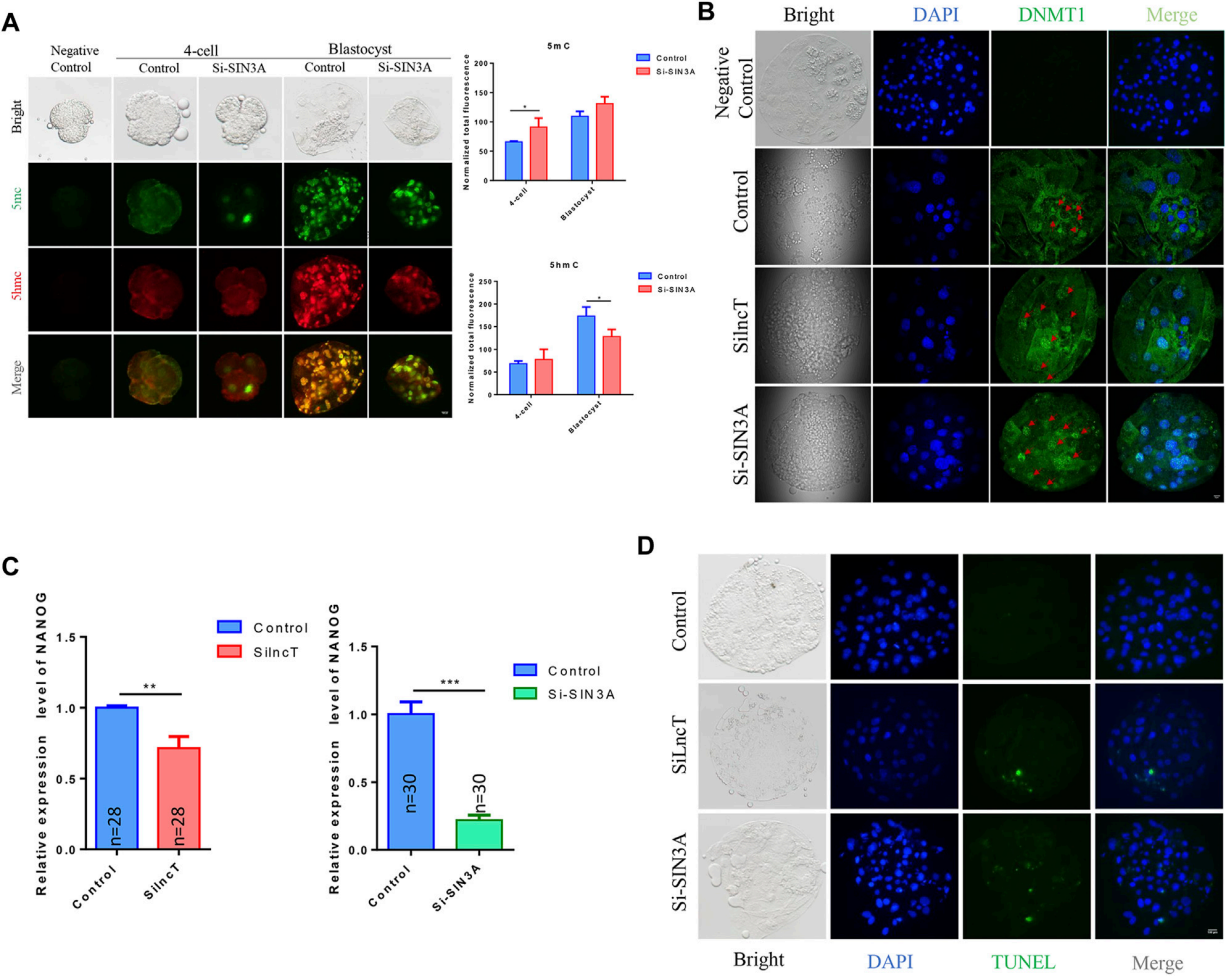
*SIN3A* affected DNA methylation and induced apoptosis in embryos. **(A)** Immunofluorescence of 5mC/5hmC in 4-cell and blastocyst stages. Fluorescence intensity analysis was in the right of the image, *p < 0.05. The experiment was repeated three times independently with 5–8 embryos/stage/group/replicate. Negative control embryos were stained without treatment with 4N-HCI and Tris-HCI. **(B)** Immunofluorescence of DNMT1 in the blastocyst; the red arrow indicated the location of the nucleus. The experiment was repeated three times independently with 5–8 embryos/stage/group/replicate. Negative control embryos were stained only with the secondary antibody. **(C)** qPCR analysis of NANOG in the control group, *lncT*-KD group, and *SIN3A*-KD group (4-cell stage, *n* = 28 or *n* = 30); **p < 0.01 and ***p < 0.001. The experiment was independently repeated three times. **(D)** TUNEL stain of the control group, *lncT*-KD group, and *SIN3A*-KD group. The green color indicated apoptosis cells. The experiment was repeated three times independently with 5–8 embryos/stage/group/replicate.

To further confirm the key genes that *lncT* regulated in embryonic development, we evaluated the top changes in genes between the two groups. As shown in Figure 6A, the heatmap showed the top 10 downregulated genes and 10 upregulated genes. Also, *SIN3A* was observed as the significantly downregulated gene after *lncT* siRNA injection (*p < 0.05) (Figure 6B). Thus, we speculated that *lncT* regulated early embryonic development partly *via* modulation of *SIN3A* expression in pigs. We selected a siRNA to interfere with the expression of *SIN3A*, as shown in Figure 6C, and *SIN3A* was significantly decreased after S1 injection (***p < 0.001). Next, we tested if the embryos had a phenotype as *lncT* depletion by knocking down *SIN3A*. As shown in Figures 6D,E, blastocyst formation was severely affected in embryos due to deficiency of *SIN3A* (**p < 0.01). Meanwhile, we detected the expression of 5mC/5hmC, as shown in Figure 7A, and the modification of 5mC in the 4-cell stage was increased after knockdown of *SIN3A* (*p < 0.05); although there was no significant difference in 5mC at the blastocyst stage, the modification of 5hmC was decreased in the *SIN3A*-KD blastocyst (*p < 0.05). The previous study reported the KD of *SIN3A*-induced DNMT1 in the nucleus (Zhao et al., 2019). Thus, we detected the expression of DNMT1; as expected, DNMT1 was mainly distributed in the nucleus in the *SIN3A*-KD group compared to the control group. Meanwhile, it was also mainly distributed in the nucleus in the *lncT*-KD group (Figure 7B). In addition, the pluripotent genes were reduced after the injection of siRNA of *lncT* (**p < 0.01) and *SIN3A* (***p < 0.001) (Figure 7C). Also, increased apoptosis was found in all interference groups (Figure 7D). These results indicated *lncT* and *SIN3A* regulated global 5mC modification and DNMT1 protein abundance and localization, and *lncT* regulated porcine early embryonic development partly through the regulation of *SIN3A* expression.

## Discussion

With the development of genomics, more and more studies have shown that non-coding RNA plays an important regulatory role in embryonic development. Wang et al. (2016) reported that *lncGET* and *lin28* are critical for mouse embryonic development beyond the 2-cell stage. *Lnc*-*137* regulates zygotic genome activation by regulating histone methylation, which is essential for early embryonic development in goats (Deng et al., 2018). However, the role of lncRNA in porcine preimplantation embryo development is rarely reported. In our study, we performed a comprehensive analysis of porcine lncRNAs based on RNAseq data on *in vivo* fertilized embryos and selected some lncRNAs that are highly expressed from the 4-cell stage. The expression pattern of these lncRNAs was consistent with the time of gene activation, and we speculated that this part of lncRNA played a role in regulating gene expression. *LncT* was one of them and highly expressed in germ organs. We first reported that *lncT* had a positive role in regulating embryonic development, and the knockdown of *lncT* inhibited cleavage and blastocyst rates. Also, the mechanism was the alteration of histone modification and DNA methylation modification; meanwhile, the downstream gene *SIN3A* also affected embryonic development.

Epigenetic modification is important in regulating porcine embryonic development. As we all know, aberrant histone modification and DNA methylation modification are considered the major reason for the developmental failure of parthenogenetic embryos and somatic cell nuclear transfer (SCNT) embryos (Hou et al., 2016; Yang et al., 2019). Many studies have found that histone methylation has a crucial role in early embryonic development and pluripotency maintenance of stem cells (Boyer et al., 2006; Sridharan et al., 2013). Shao et al. (2008) reported that the increased expression of H3K4me2 at the 2-cell stage causes abnormal activation of embryonic gene expression and further reduction of developmental efficiency in mice. The H3K4me3 expression level is decreased and then increased in the early development of the human, mouse, and porcine naturally fertilized embryos and then symmetrically distributed in ICM and TE of blastocysts (Gao et al., 2010; Zhang et al., 2012; Cao et al., 2015). However, the regulation of lncRNA in embryonic epigenetics remains largely unexplored. Yang et al. (2020) reported that lnc2193 decreases the expression level of H3K4me3 and H3K9me3, thus affecting the porcine oocyte maturation. Deng et al. (2021) have found that lnc_3712 regulates H3K4me3 and affects the development of goat embryos *via* repressing KDM5B. In our study, we analyzed the expression profiles of representative epigenetic modification enzymes from the sequencing data and found that *KDM5B*, *KDM5C*, and *SETD2* were upregulated after *lncT* knockdown. Meanwhile, we observed a decrease in H3K4me3 and H3K4me2 levels at the 2-cell stage and 4-cell stage, which confirmed the knockdown of *lncT* could disturb the epigenetic modification such as H3K4 and H3K9.

DNA methylation is associated with histone modifications, particularly the absence of histone H3 lysine 4 methylation (H3K4me0) and the presence of H3K9 methylation (Hashimoto et al., 2010). A previous study has shown that the Mll1 knockdown prevents H3K4me3 and DNA 5hmC changes in the rat hippocampus (Webb et al., 2017), while our previous study indicates a positive correlation between H3K4 methylation and DNA methylation in SCNT embryos (Zhang et al., 2018). In this study, we found an increased expression level of 5mC and decreased expression level of H3K4me3 in *lncT*-KD embryos, which was different from the results in SCNT embryos, while the increase in the DNA methylation level certainly would be the barrier to zygotic genes and cellular reprogramming, which is consistent with the phenotype we observed.

*SIN3A* is a scaffold component of the chromatin repressive complex Sin3/histone deacetylase (HDAC) (Zhao et al., 2019). It is reported that the Sin3 complex promotes transcription or inhibits transcription in different physiological states (Laugesen and Helin, 2014) (Icardi et al., 2012), while little research has been conducted on the function of *SIN3A* during embryonic development. A previous study indicates that *SIN3A* deficiency in mouse early embryos causes embryonic block at the morula stage, which is mediated through the regulation of *Hdac1* (Zhao et al., 2019). Luo et al. (2021) reported that *SIN3A* is required for porcine early embryonic development, and the knockdown of *SIN3A* inhibits blastocyst formation through the regulation of *CCNB1* expression. Zhao et al. (2019) indicated that *SIN3A* regulated the development progress in mice, and the KD of *SIN3A* could induce DNMT1 to the nucleus. However, little is known about lncRNAs in the regulation of *SIN3A* during embryonic development. Our results showed that *SIN3A* was the most downregulated gene among the top 10 downregulated genes after *lncT* knockdown. We observed a severe blastocyst formation defect in *SIN3A-*KD embryos. The amount of 5mC and DNMT1 was increased relatively in *SIN3A*-KD embryos, which was consistent with previous reports (Zhao et al., 2019). Meanwhile, we observed the increased expression level of 5mC and the movement of DNMT1 to the nucleus in *lncT-*KD embryos. It is suggested that interfering RNA can interfere with the level of DNA methylation modification, thus hindering the correct expression of genes and affecting the development of embryos. However, we have not found a direct regulatory relationship between *lncT* and *SIN3A*, and further research is needed.

The expression of pluripotency genes is crucial for the development of preimplantation embryos (Hanna et al., 2010). A previous study reported the role of *SIN3A* in regulating the pluripotent embryonic cell cycle, and *MYC* and *E2F* targets in *SIN3A*-null ICMs are downregulated (McDonel et al., 2012a). Patrick et al. reported the deletion of *SIN3A* in MEFs results in a profound growth defect, significant G2/M accumulation, and increased apoptosis (McDonel et al., 2012b), while Zhao et al. (2019) did not detect changes in apoptosis in mice embryos. They attribute this difference to stage dependence or environment dependence. In our study, we found that *NANOG* was decreased in both siRNA groups, suggesting the regulation function of *lncT* and *SIN3A* on *NANOG*. In addition, the number of apoptotic cells was increased, to some extent, in blastocysts after siRNA injection. Our results indicated that the knockdown of *lncT* and *SIN3A* affected embryo development *via* inhibiting the expression of *NANOG* and inducing apoptosis.

Our results first demonstrate that *lncT* is one of the key lncRNAs regulating embryonic development. It may affect embryonic development by regulating epigenetic modifications, such as H3K4me3, H3K4me2, H3K9me3, and DNA methylation modification. In addition, *SIN3A* is one of the key genes affected by *lncT*, and both of them can affect the pluripotency gene *NANOG* and induce more cell apoptosis. These findings fill in the deficiency of lncRNA research in porcine embryonic development and provide theoretical support for biomedical and animal husbandry production.

## Data Availability

The datasets presented in this study can be found in online repositories. The names of the repository/repositories and accession numbers can be found at: https://www.ncbi.nlm.nih.gov/, GSE207258

## Abbreviations

LncRNA, long non-coding RNA; IVV, *in vivo* fertilization; IVF, *in vitro* fertilization; KD, knockdown; ZGA, zygotic genome activation; DEGs, differentially expressed genes; IVM, *in vitro* maturation; COCs, cumulus–oocyte complexes; PGM, porcine gamete medium; IF, immunofluorescence; PVP, polyvinylpyrrolidone; KEGG, Kyoto Encyclopedia of Genes and Genomes; FDR, false discovery rate; FC, fold change; SCNT, somatic cell nuclear transfer.
