# Supplementary material for: LncRNA affects epigenetic reprogramming of porcine embryo development by regulating global epigenetic modification and the downstream gene SIN3A
Source: Front Physiol. 2022 Sep 16;13:971965. doi: 10.3389/fphys.2022.971965 (PMC9523245; doi:10.3389/fphys.2022.971965)
Supplement: Supplementary file 1 [file Table1.DOCX]

LncT 2038bp

TCTTGGGTTGAGTGTTGTGCGCAGCGCGCCTGCTCCGGAGCGGAGCTGACCAGGCAGCAGTTGAGCATCAGAAGCTCTGGTCCTGAGCGCTGGGTGCGAGCACCGTGCGAGCAAGTGATGCCGGACTGAACGCAAAGGAAAAAGCAAAAGGTGCGGCTCCTACTGCCAGGTGACCCGGCCTCTCCACAGTTACCTGGCTCAGTACTTGGCTGCCTGAGCCACAGGACGCGCGTCAGGACTGCTTCCCCTGGAATTTGTAATCGGAACAGGGAGAGATGCTGAGGGACCTGGGCTCCCCTGACTTGCCCAGTGTGCTCTGGGGCCACGTCTGTGTCATAGCTCGCTCAGGACACAGCACAGGAACTGGGGCGTGATGCCCAGAGCTGACCAGGGAGGAGCACGCACACGAGACGCAAAGCTGTTGGAGGCAGGAGAGCTGCTGGACGAATGGTAACTGGCTTAGTATTCCAGAGAATTCCGTGAAGTTCTAGGTCTGTGGGGGTGGGGAGGGTGAGCACATTCTTGCCTGAGAACCCCAGAAATGGCCAACAATGGGAACTCTTAAGGATTCCCTGGGGTAAGAAGGACCTTGCAAGGAGCACTTAGACCTGAGGTCTGTCCATCAGTTCGTGCCAGTGGGTACCCGCCCCTTCTGATCCCAGGAAAAGAGGAGAGGTTTCCTCTGAGGGTTGGAAGTAGAGGACCTGGCCTCAGAGATCCCAGCCTAACCTGGGCTGCAGGTCCTGAACTGAAAACAGGGAGTGGGAAGGAATGTTGATTACTGAGCAGGGAAATGCCCGGTGTCTTCCTGACTCTGCTTCTAGAATCCCAGCAGTTAACATCTGGCCAGCACCTGGAGGGTTTGTCACAGAGAGGGTTGGATGCCACCTAAGATGTGGGGTGCCCACGTCTTACGACTTGGGACTTGGGACCTTGGACCCCTACTGGGAGACCTGTGGGTGGATAAGCTCTGTCTGTCCTGCCAGCAGCATTTGAATCTCACTCTTCAATGTGAATAAATAGCCAAGATCACCTGACCTTGGAGGGAAGGTTCCCAGATGAGATGGTGATAAACACCAACAGAAAGAGGAGTTCTGCGCAGGGAGAGACAGTGCAGAGAGCGGAGAACGTCGGAGAAGCAGAGACGAGAGGCTAGCCCCCAAGCGTGACTTTGCTGGCTTCCCAGTGTTCACGTCCTCTGGAGTCCCTGCCTTGCCCATCCCTGGTTTCTGTGGCACAACCCTCTGTCTGCCAAAGAGCCCCCAGGTGCTGAAGATGCTATGAATGTGTTTCCTTCCCTTCTGACCAAAGGGGTGCCTGCCCCCACCCCTCATCCTGCTGACCCCAGCAGCTTGGAAGAAGAGGCCTCCCCTGGGTTAGCAGTCGGCCCAGCATCTCTGCCAAGTGACCCTGGCAGCTGCAGCCTCCCCAAGCCCCCAGGGAACCAGGGGCATGTCCAAAAGGCTCCATCTGAGGCTGGCTCAGCTCCCGGCTGCCCTCTTGCTGGCAGGGGATGGAGCCAATTGGCCCTGTGTCTGCATTGGGAGCTGTGAGCCCACGCTTGTTTGTAGCCGTCACCAGTGTCCTCTCTCTTTCGCTGGACCAGGCAGGGCTCAGGCTCCTTTGTGTCCTCTCTCCTCCATGTCCAGAAGCTCTGGGGCCACGGTCTTGATTAACCTAACCCTCTTCAAATGAATAAATAATATGTCTCTAAATAGGACTTTAAATATCTGAACAGCAGCTGTGAACGTTCATTCAGTTGCTGCACGTTTATGAAGCGTTTTGTCTCTGCCTATAGGACTTAGTCCTTTTTTGTAATTAGCGCAGATGTATTTTCTAGGTTCTAATATCAAATACCAATAATTTCATAAATGGGTCGAAAAGAATGAGGTTGTTTTGATAACGTGAATATTTGAAGTAAGGGTTTGACGACTGCGTGATCAGCAAGTCATCATCAAGTTGATGGGGTTTGTCATCAACATCAGGGCATTTTGAAGAGTGAAACCAGGCACGTGACACGAAACTGGGCACGTGACAT

>XR_308890.3 PREDICTED: Sus scrofa uncharacterized LOC102165808 (LOC102165808), transcript variant X1, ncRNA

TCTTGGGTTGAGTGTTGTGCGCAGCGCGCCTGCTCCGGAGCGGAGCTGACCAGGCAGCAGTTGAGCATCAGAAGCTCTGGTCCTGAGCGCTGGGTGCGAGCACCGTGCGAGCAAGTGATGCCGGACTGAACGCAAAGGAAAAAGCAAAAGGTGCGGCTCCTACTGCCAGGTGACCCGGCCTCTCCACAGTTACCTGGCTCAGTACTTGGCTGCCTGAGCCACAGGACGCGCGTCAGGACTGCTTCCCCTGGAATTTGTAATCGGAACAGGGAGAGATGCTGAGGGACCTGGGCTCCCCTGACTTGCCCAGTGTGCTCTGGGGCCACGTCTGTGTCATGTATGATGGGAAGACGGGACAGAGGGGGGCAGAGGGGGGCAGACGGAGTTCCTGGGCGGAGAGAAGCAGGAGCTTCCGGCTCAGTGTGGCAGGCTAGACCCCCCGGTTATCGCCACTCCCTTGGCAGAGCTCGCTCAGGACACAGCACAGGAACTGGGGCGTGATGCCCAGAGCTGACCAGGGAGGAGCACGCACACGAGACGCAAAGCTGTTGGAGGCAGGAGAGCTGCTGGACGAATGCATTTGAATCTCACTCTTCAATGTGAATAAATAGCCAAGATCACCTGACCTTGGAGGGAAGGTTCCCAGATGAGATGGTGATAAACACCAACAGAAAGAGGAGTTCTGCGCAGGGAGAGACAGTGCAGAGAGCGGAGAACGTCGGAGAAGCAGAGACGAGAGGCTAGCCCCCAAGCGTGACTTTGCTGGCTTCCCAGTGTTCACGTCCTCTGGAGTCCCTGCCTTGCCCATCCCTGGTTTCTGTGGCACAACCCTCTGTCTGCCAAAGAGCCCCCAGGTGCTGAAGATGCTATGAATGTGTTTCCTTCCCTTCTGACCAAAGGGGTGCCTGCCCCCACCCCTCATCCTGCTGACCCCAGCAGCTTGGAAGAAGAGGCCTCCCCTGGGTTAGCAGTCGGCCCAGCATCTCTGCCAAGTGACCCTGGCAGCTGCAGCCTCCCCAAGCCCCCAGGGAACCAGGGGCATGTCCAAAAGGCTCCATCTGAGGCTGGCTCAGCTCCCGGCTGCCCTCTTGCTGGCAGGGGATGGAGCCAATTGGCCCTGTGTCTGCATTGGGAGCTGTGAGCCCACGCTTGTTTGTAGCCGTCACCAGTGTCCTCTCTCTTTCGCTGGACCAGGCAGGGCTCAGGCTCCTTTGTGTCCTCTCTCCTCCATGTCCAGAAGCTCTGGGGCCACGGTCTTGATTAACCTAACCCTCTTCAAATGAATAAATAATATGTCTCTAAATAGGACTTTAAATATCTGAACAGCAGCTGTGAACGTTCATTCAGTTGCTGCACGTTTATGAAGCGTTTTGTCTCTGCCTATAGGACTTAGTCCTTTTTTGTAATTAGCGCAGATGTATTTTCTAGGTTCTAATATCAAATACCAATAATTTCATAAATGGGTCGAAAAGAATGAGGTTGTTTTGATAACGTGAATATTTGAAGTAAGGGTTTGACGACTGCGTGATCAGCAAGTCATCATCAAGTTGATGGGGTTTGTCATCAACATCAGGGCATTTTGAAGAGTGAAACCAGGCACGTGACACGAAACTGGGCACGTGACAT
