# Supplementary figures and images for: LncRNA affects epigenetic reprogramming of porcine embryo development by regulating global epigenetic modification and the downstream gene SIN3A
Source: Front Physiol. 2022 Sep 16;13:971965. doi: 10.3389/fphys.2022.971965 (PMC9523245; doi:10.3389/fphys.2022.971965)

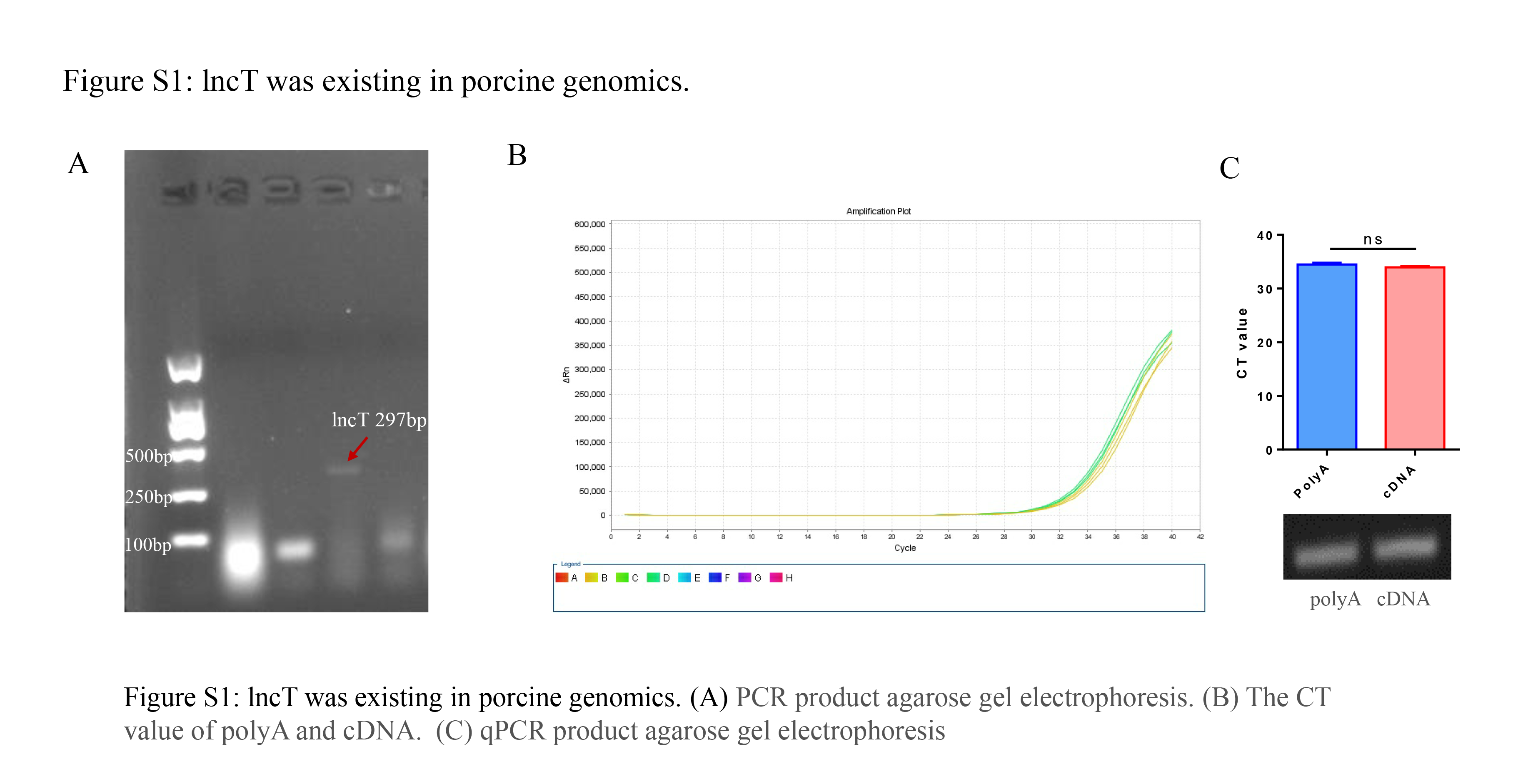

Supplement: Supplementary file 3 [file Image1.TIF]
